# Investigation of recycled materials for radiative cooling under tropical climate

**DOI:** 10.1515/nanoph-2023-0593

**Published:** 2023-10-30

**Authors:** Di Han, Jipeng Fei, Man Pun Wan, Hong Li, Bing Feng Ng

**Affiliations:** School of Mechanical and Aerospace Engineering, Nanyang Technological University, 50 Nanyang Avenue, Singapore 639798, Singapore

**Keywords:** recycled polymer, sub-ambient radiative cooling, solar reflectance, tropical climate

## Abstract

As a sustainable alternative to using virgin polymer, we propose the use of recycled polymer for the fabrication of passive radiative cooling materials to tackle both the increasing demand for cooling systems and to upcycle plastic waste. Using recycled acrylic sheets as the binder for BaSO_4_ thorough sol–gel method, a sustained 1.2 °C sub-ambient temperature during daytime and 3 °C sub-ambient temperature during nighttime was achieved under the hot and humid conditions of the tropical climate. The coating achieved 97.7 % solar reflectance and 95 % infrared emittance. Separately, when porosity is introduced to recycled acrylic sheets through a phase inversion method, a near-ambient temperature during noontime and sustained sub-ambient temperature of 2.5 °C during nighttime was achieved (with 96.7 % solar reflectance). Comparable performances are also obtained using recycled polyvinyl chloride (PVC) pipe and expanded polystyrene (EPS) foam.

## 1 Introduction

Conventional methods to indoor-cooling involve the active use of air-conditioning systems which leads to high energy demands. On the other hand, passive radiative cooling (PRC) systems provide free cooling by regulating the amount of heat transmitted indoors through a combination of reflection and emission strategies [1, 2]. It works by transferring radiation heat from an object on the surface of the Earth to the cold outer space (around 3 K) [3–5] through the atmospheric window. Even though the earth’s atmosphere (comprised of a mixture of gases including N_2_, O_2_, CO_2_ and water vapor) absorbs heat, it becomes transparent to thermal radiation in the wavelength of 8–13 µm for heat from terrestrial objects to be directly emitted to the outer space (ultimate heat sink) [6, 7]. Accompanied with reflection of solar radiation in the wavelength of 0.3–2.5 µm, materials exhibiting selective optical properties are able to enhance cooling and even reach sub-ambient temperatures [8–10]. To passive daytime radiative cooling (PDRC), studies have shown that a reflectivity of 95 % and above in the solar spectrum is required for effective cooling performances [8]. However, in a region with tropical climate, the criteria of solar reflectivity becomes more stringent due to the higher solar intensity, ambient temperature and relative humidity [11], where over 97 % solar reflectivity is needed to minimise heat gain from the sun during daytime [12, 13].

Recently, different forms of effective PDRC materials such as paints and films have been proposed for real world applications [14–16]. Among them, polymer-based PDRC materials have shown excellent cooling performances alongside their low cost and high potential for scalability [17]. These organic polymers, including poly(vinylidene fluoride-co-hexafluoropropylene) (PVDF-HFP), polyvinylidene fluoride (PVDF) polymethyl methacrylate (PMMA), polystyrene (PS) and polyvinyl chloride (PVC), have displayed strong infrared (IR) emission due to their intrinsic vibrations within 8–13 μm [9, 18], [19], [20], [21]. To achieve high solar reflectivity, white pigment nanoparticles (NPs) with strong Mie scattering efficiency within 0.3–2.5 µm can be embedded within the polymers [22]. Separately, hierarchically porous polymeric structures have also proven to be effective in achieving high solar reflectance and IR emittance concurrently [9, 23, 24]. However, almost all studies thus far have considered the use of virgin polymeric materials, which taps on limited natural resources and potentially leading on to increased landfill burden. In fact, the global demand for plastics has become an environmental concern in recent years as the amount of plastic being disposed annually has seen a steep upward trend [25]. Hence, a possible solution to the sustainable development of PDRC is to substitute virgin polymer with their recycled counterparts during the manufacturing process. Moreover, earlier research have proven that recycled plastics can be modified to provide similar properties and performance when compared to virgin plastics [26, 27], which provide a possibility for the development of recycled radiative coolers [28–31].

In this work, we investigate the viability of using recycled polymers from commercial plastic waste products to create polymeric coatings through sol–gel and phase inversion methods under the tropical weather testing. Using recycled acrylic sheets as the binder for BaSO_4_ thorough sol–gel method, a sustained 1.2 °C sub-ambient temperature during daytime and 3 °C sub-ambient temperature during nighttime was achieved under the hot and humid conditions of the tropical climate. Separately, when porosity is introduced to recycled acrylic sheets through a phase inversion method, a near-ambient temperature during noontime and sustained sub-ambient temperature of 2.5 °C during nighttime was achieved. Other commercial plastics such as PVC pipe and EPS foam also exhibited potential for replacing virgin polymers while providing comparable cooling performances.

## 2 Materials and methods

### 2.1 Preparation of radiative cooling samples

#### 2.1.1 Sol–gel method

**Figure 1:**
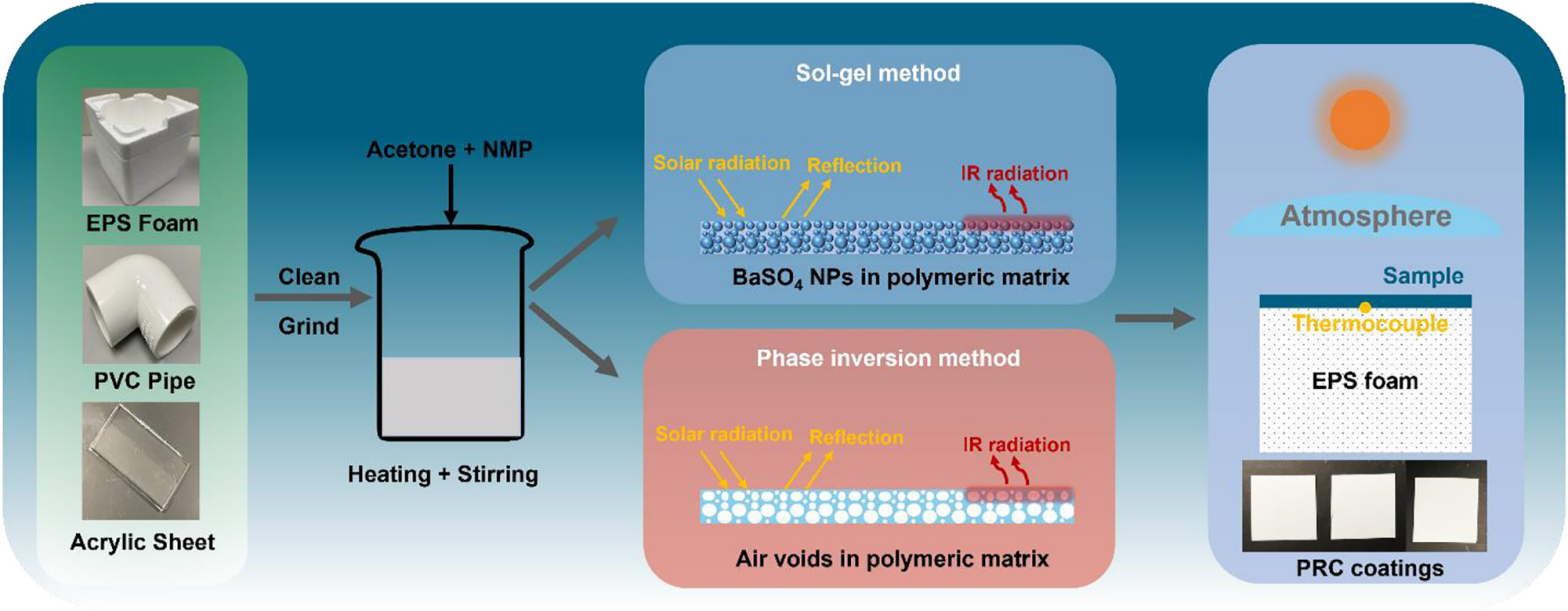
Recycled passive radiative coolers using sol–gel and phase inversion methods, including set-up for radiative cooling performance and optical image of PRC coatings.

The recycled polymeric products: expanded polystyrene (EPS) foam, polyvinyl chloride (PVC) pipe and acrylic sheet) are cleaned and grinded into small pieces using the grinder. Subsequently, small pieces of the recycled polymer were added to a mixture of *N*-methyl-2-pyrrolidone (NMP) and acetone as shown in Figure 1. The combined mixture was then heated to 80 °C on a heating plate and stirred until the polymer completely dissolves into the solution. BaSO_4_ powder (Sigma-Aldrich) was dispersed into the polymer-solvent system and the stirring process was done for another 30 min at 80 °C to ensure homogenous mixing of the BaSO_4_ particles within the polymer matrix. The coating precursor was subsequently coated onto a polished aluminium plate using a blade coating machine for solvent evaporation. The same steps and solvents were used for the preparation of all recycled plastics in this study and the mass ratio of BaSO_4_ and polymer was 15:1 to achieve a highly reflective and stable surface without any cracks.

#### 2.1.2 Phase inversion method

Once a homogeneous solution is obtained as shown in Figure 1, it is immediately coated onto an aluminium sheet by a blade coating machine. After air drying for 10 min, the sample was moved into a water bath for phase inversion process to obtain a freestanding film with air voids in the polymeric matrix [19].

### 2.2 Characterizations of radiative cooling performance

The characterizations of radiative cooling performance were performed on the rooftop of Academic Block North in Nanyang Technological University, Singapore. As shown in Figure 1, the sample was facing the sky and thermal insulated by EPS foam with the same size. The surface thermocouple (SA3-K-120, Omega) was attached to the centre of the sample at the bottom surface for measurement of the surface temperature. A nearby weather station was employed to monitor the weather conditions including ambient air temperature, relative humidity, solar irradiance and atmospheric radiation. For the cooling power test, a polyimide film heater powered by a constant current source was attached to the bottom surface of the sample. To obtain effective cooling power, the sample was heated to match the ambient air temperature.

### 2.3 Characterizations of optical properties

The spectrum solar reflectance within the wavelength of 0.3–2.5 μm was measured using a UV–VIS-NIR spectrometer (PerkinElmer-Lambda 950). The spectrum IR reflectance within the wavelength of 2.5–20 μm was measured with a Fourier transform infrared spectrometer (PerkinElmer Spectrum 3) connected to a gold-coated integrating sphere (PIKE Mid-IR IntegratIR). A field-emission scanning electron microscope (JEOL 7600L) was employed to obtain the SEM images.

## 3 Optical and radiative cooling performance of BaSO_4_ coating

**Figure 2:**
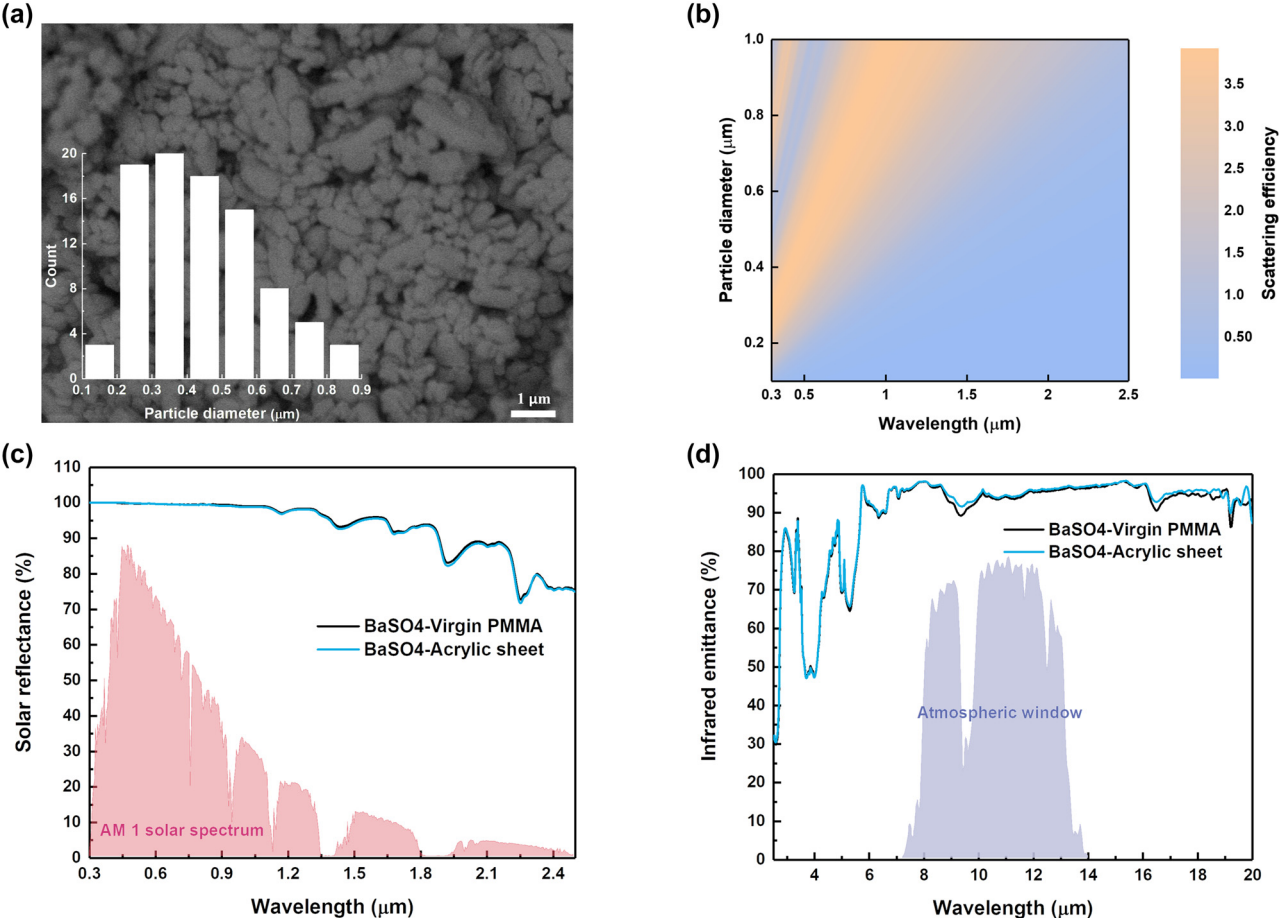
Optical performance of the radiative cooling sample using sol–gel method. (a) SEM image of the sample. Inset is particle size distribution. (b) Scattering efficiency of the BaSO_4_ NPs within the solar spectrum from FDTD simulation. (c) Measured solar reflectance of the BaSO_4_ coating using the recycled acrylic sheet and virgin PMMA (background is AM1 solar spectrum [32]). (d) Measured infrared emittance of the BaSO_4_ coating using the recycled acrylic sheet and virgin PMMA (background is atmospheric window).

To achieve high solar reflectance, BaSO_4_ particle was added to the polymers during the sol–gel fabrication process. As shown in Figure 2(a), the BaSO_4_ particles has a broad particle diameter range from 0.1 to 1 μm, and form a dense structure after binding by the recycled acrylic polymer. Owing to the comparable size of the particles with the wavelength of sunlight, strong Mie-scattering is achieved to highly reflect solar heat. Here, the finite-difference time-domain (FDTD) simulation (details in Supporting Information, Note 1 and Figure S1) was employed to calculate the scattering efficiency of BaSO_4_ particles as a function of diameter from 0.1 to 1 μm according to Mie theory. As shown in Figure 2(b), the particles with diameter of around 0.1–1 μm could generate relative-high scattering efficiency within the wavelength of 0.3–1.5 μm, which means that it can strongly reflect the majority of solar energy. The solar reflectance of the BaSO_4_ particles with recycled acrylic polymer as binder could reach around 97.7 %, which is almost the same level as that with virgin PMMA as shown in Figure 2(c). Both coatings also have similar infrared emittance, with an average emittance of 95 % within 8–13 μm as shown in Figure 2(d). Likewise, for recycled PVC pipe and EPS foam with BaSO_4_ particles, the solar reflectance and IR emittance (as shown in Figure S2(a) and (b)) are comparable to the one with recycled acrylic polymer as binder.

**Figure 3:**
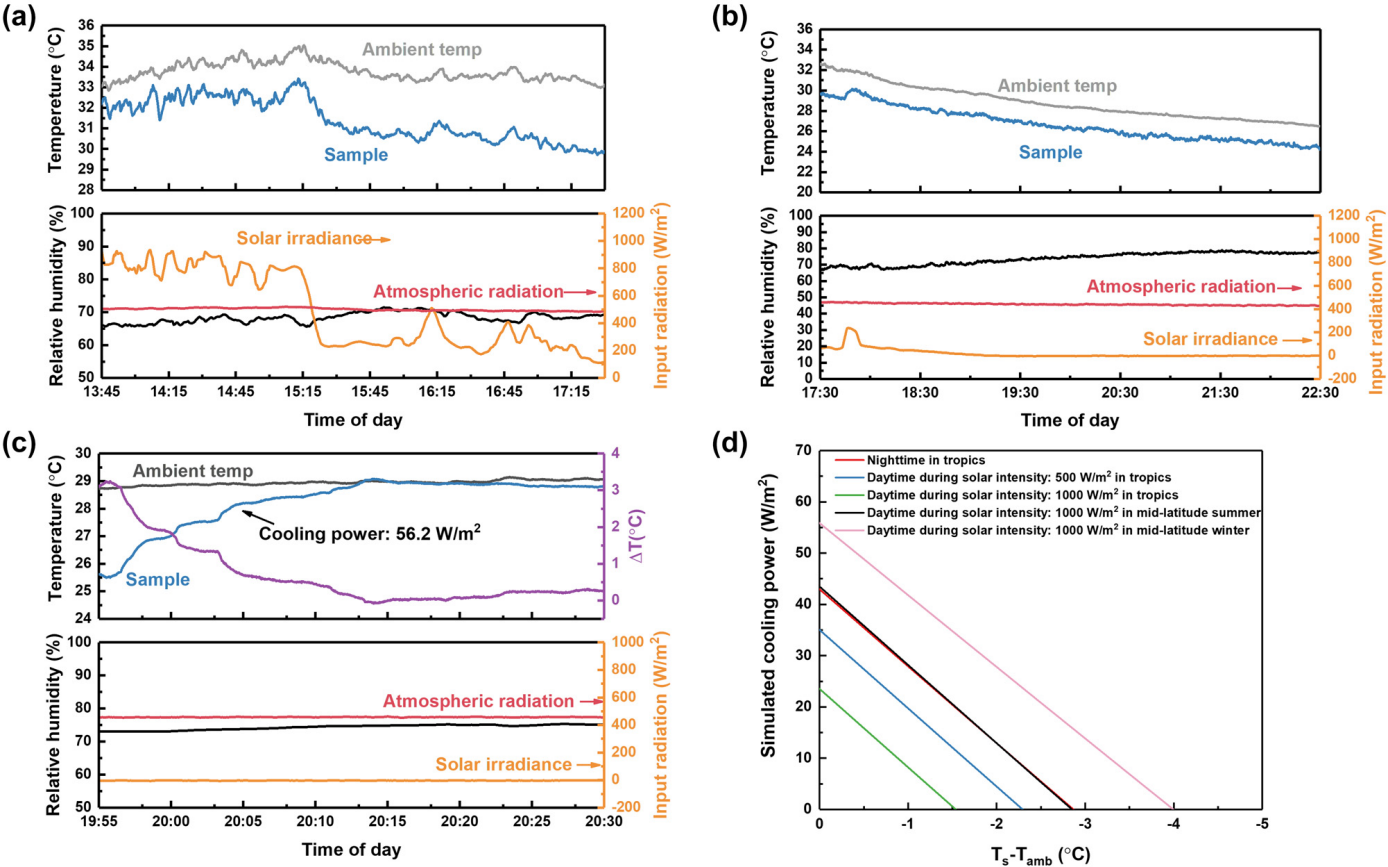
Radiative cooling performance of the sample using sol–gel method. (a) Measured surface temperature during daytime with local ambient air temperature, humidity, solar irradiance and atmospheric radiation. The sunlight was blocked by the clouds in the sky after 15:15 on that day (08-Sep-2023). (b) Measured surface temperature during night-time with local ambient air temperature, humidity, solar irradiance and atmospheric radiation (08-Sep-2023). (c) Measured surface temperature during night-time with heater turned on at time 19:56 for the sample to match ambient temperatures for measurements of cooling power (56.2 W/m^2^) (11-Sep-2023). (d) Simulated radiative cooling power with different solar intensity and climate.

Next, the radiative cooling performance of the BaSO_4_ coating with recycled acrylic polymer is examined under Singapore’s tropical climate. As shown in Figure 3(a), consistent sub-ambient temperature of 1.2 °C was achieved during noontime when the solar intensity is 900 W/m^2^ and atmospheric radiation is 500 W/m^2^. During night-time without solar heat gain, the temperature of the coating was 3 °C below ambient as shown in Figure 3(b). This amount to an effective cooling power of around 56.2 W/m^2^ as shown in Figure 3(c) and further simulations of radiative cooling power is detailed in Supporting Information, Note 2 and shown in Figure 3(d). Moreover, the cooling performance of BaSO_4_ coating using virgin PMMA and other recycled polymer (PVC pipe and EPS foam.) as the binder showed similar surface temperatures during the 24 h continuous test (details in Figure S2(c)).

## 4 Optical and radiative cooling performance of porous film

**Figure 4:**
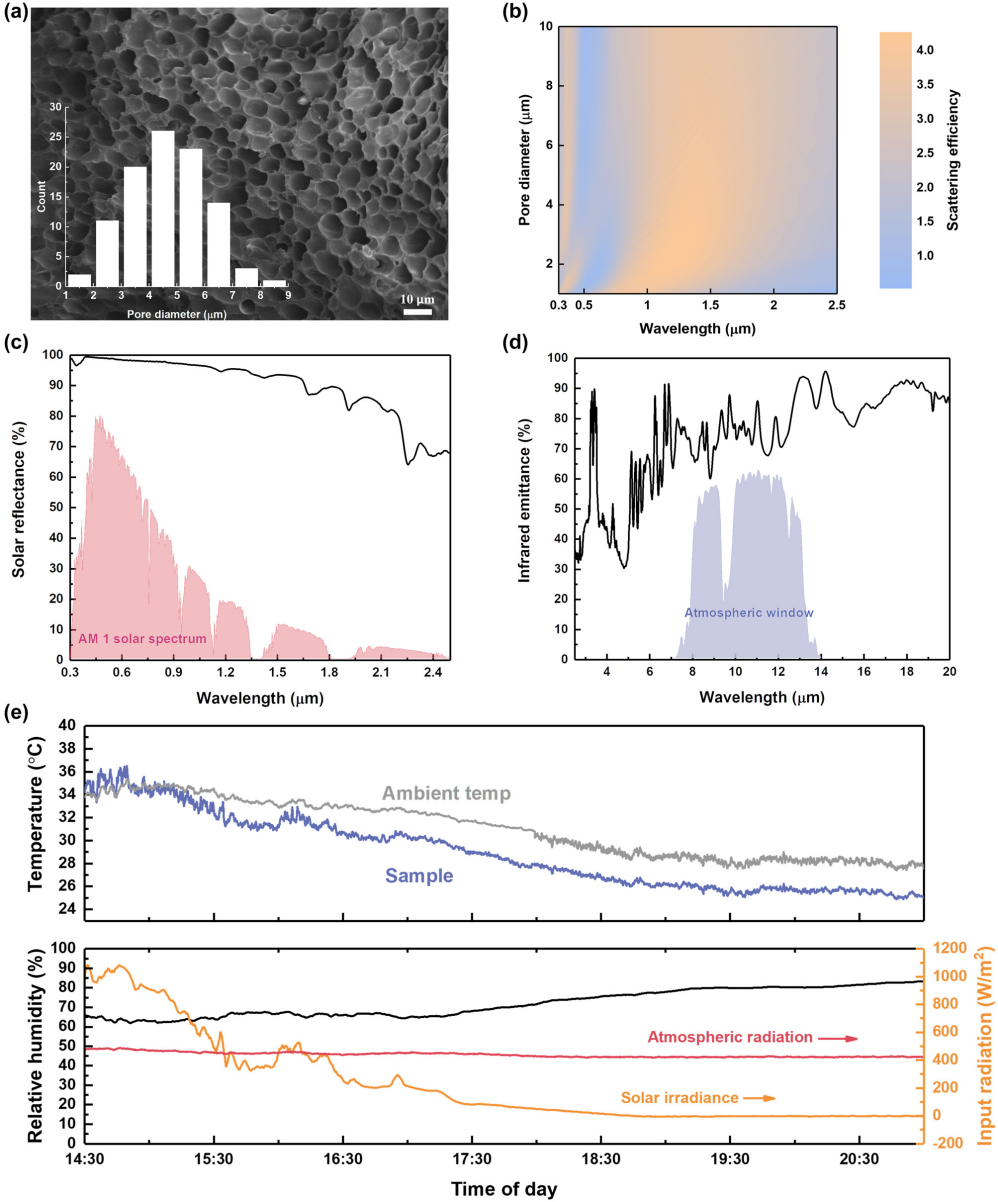
Optical and radiative cooling performance of the radiative cooling sample using phase inversion method. (a) SEM image of the sample. Inset is pore size distribution. (b) Scattering efficiency of the air pores within the solar spectrum from FDTD simulation. (c) Measured solar reflectance of the porous film using the recycled acrylic sheet (background is AM1 solar spectrum [32]). (d) Measured infrared emittance of the porous film using the recycled acrylic sheet (background is atmospheric window). (e) Outdoor radiative cooling performance under tropical climate: measured surface temperature with local ambient air temperature, humidity, solar irradiance and atmospheric radiation (27-Oct-2022).

Using the phase inversion method, porous films with nano–micro air pores are fabricated using recycled acrylic sheets. As shown in Figure 4(a), the air pores inside the film comprise of a broad size distribution with diameter ranging from 1 to 10 μm. From the FDTD simulations (details in Supporting Information, Note 1 and Figure S1) and as shown in Figure 4(b), relative high scattering efficiency can be achieved across the solar spectrum for pore diameters between 1 and 10 μm. Overall, the solar reflectance of the porous film was 96.7 % as shown in Figure 4(c), with an infrared emittance of 75 % in the atmospheric window as shown in Figure 4(d). The radiative cooling performance of the freestanding porous film under tropical climate was also evaluated and is shown in Figure 4(e). The surface temperature of the sample could reach near-ambient temperatures during noontime where solar intensity and atmospheric radiation are above 1000 W/m^2^ and 400 W/m^2^ respectively. During night-time, sustained sub-ambient temperatures of 2.5 °C can be achieved. For the remaining two recycled polymers (PVC pipe and EPS foam), owing to comparable levels of optical properties, their porous films could achieve similar radiative cooling performance compared with the one using recycled acrylic sheet (details in Figure S3).

## 5 Conclusions

The viability of using recycled polymers (acrylic sheet, PVC pipe and EPS foam) as a substitute for virgin polymer in radiative cooling is investigated in this study. Through sol–gel method, BaSO_4_ coating with recycled acrylic sheet as the binder exhibited 97.7 % solar reflectance and 95 % IR emittance. Under the harsh conditions of the tropical climate that is characterized by high solar irradiance and high humidity, the coating is able to achieve sustained sub-ambient temperatures of 1.2 °C and 3 °C during daytime and nighttime, respectively. Through phase inversion method, the solar reflectance of the porous film using recycled acrylic sheet was 96.7 %. A near-ambient temperature during noontime and sustained sub-ambient temperature of 2.5 °C during nighttime was achieved. Similar performances could be obtained for BaSO_4_ coating and porous film using recycled PVC pipe and EPS foam.

## Supplementary Material

Supplementary Material Details
