# Supplementary material for: Investigation of recycled materials for radiative cooling under tropical climate
Source: Nanophotonics. 2023 Oct 30;13(5):593–9. doi: 10.1515/nanoph-2023-0593 (PMC11501400; doi:10.1515/nanoph-2023-0593)
Supplement: Supplementary file 1 — Supplementary Material Details [file j_nanoph-2023-0593_suppl_001.docx]

Supporting information

Investigation of Recycled Materials for Radiative Cooling under Tropical Climate

Di Han^a,1^, Jipeng Fei^a,1^, Man Pun Wan^a^, Hong Li^a^, Bing Feng Ng^a*^

*^a^School of Mechanical and Aerospace Engineering, Nanyang Technological University, 50 Nanyang Avenue, Singapore 639798*

*Corresponding author: [bingfeng@ntu.edu.sg](mailto:bingfeng@ntu.edu.sg) (Bing Feng Ng).

^1^These authors contributed equally to this work.

# Note 1 Finite-Difference Time-Domain (FDTD) simulation

Finite-Difference Time-Domain (FDTD) simulations (Ansys Lumerical version: 2023 R2.2) were employed to analyse the scattering effect of nanoparticles and air pores within the solar irradiance wavelength (0.3 to 2.5 μm). To simplify the computation process and reduce simulation time, a two-dimensional model was used and shown in Figure S1. Here, the total-field scattered-field (TFSF) source and perfect matching layer (PML) boundary conditions was used to calculate the scattering efficiency. For the nanoparticles, the diameter varies from 0.1 to 1 μm. For the air pores, the outer diameter varies from 1 to 10 μm while the wall thickness is fixed at 0.5 μm.


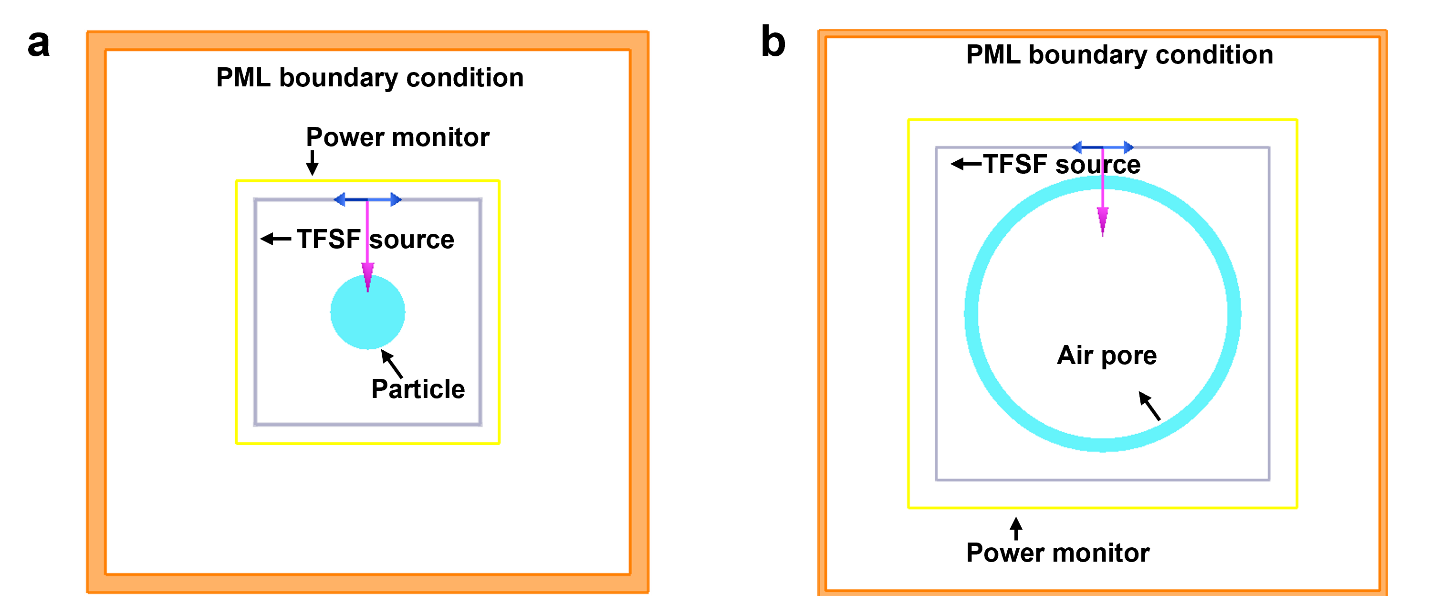


**Figure S1:** FDTD simulations. (a) Scattering effect simulation of nanoparticles. (b) Scattering effect simulation of air pores.

# Note 2 Calculation of radiative cooling power

The energy balance equation of the radiative cooling sample with a surface temperature $T_{s}$ can be expressed as:

$$\begin{aligned} P_{cool}\left( T_{s} \right)=P_{rad}\left( T_{s} \right)-P_{atm}\left( T_{amb} \right)-P_{solar}-P_{comb}\left( T_{s},T_{amb} \right)\#\left( 1 \right) \end{aligned}$$

$P_{rad}$ is the output radiative power by the surface, which can be calculated using the expression:

$$\begin{aligned} P_{rad}\left( T_{s} \right)=2\pi\int_{0}^{\frac{\pi}{2}} sin\theta cos\theta\int_{0}^{\infty} I_{B}\left( T_{s},\lambda\right)\varepsilon\left( \lambda,\theta\right)d\theta d\lambda\#\left( 2 \right) \end{aligned}$$

where $I_{B}\left( T_{s},\lambda\right)$ is the spectral radiance of a blackbody with a surface temperature of $T_{s}$ by Planck’s law which is $I_{B}\left( T_{s},\lambda\right)=\frac{2hc^{2}}{\lambda^{5}}\frac{1}{e^{hc/\lambda k_{B}T_{s}}-1}$. Here, *h* is the Planck’s constant, $k_{B}$ is the Boltzmann constant, *c* is the speed of light, $\varepsilon\left( \lambda,\theta\right)$ is the measured spectral and angular emissivity of the sample.

$P_{atm}$ is the input atmospheric radiation absorbed by the surface which can be calculated using:

$$\begin{aligned} P_{atm}\left( T_{amb} \right)=2\pi\int_{0}^{\frac{\pi}{2}} sin\theta cos\theta\int_{0}^{\infty} I_{B}\left( T_{amb},\lambda\right)\varepsilon_{atm}\left( \lambda,\theta\right)\varepsilon\left( \lambda,\theta\right)d\theta d\lambda\#\left( 3 \right) \end{aligned}$$

Where $T_{amb}$ is the ambient temperature, $\varepsilon_{atm}\left( \lambda,\theta\right)$ is atmospheric emissivity influenced by the wavelength and angle. It can be calculated by the atmospheric transmittance $t (\lambda)$ [1] in the zenith direction using $\varepsilon_{atm}\left( \lambda,\theta\right)=1-{t(\lambda)\text{ }}^{1/cos\theta}$ [2].

$P_{solar}$ is the input solar power absorbed by the surface which can be expressed by:

$$\begin{aligned} P_{solar}=\int_{0}^{\infty} \varepsilon\left( \lambda,\theta_{solar} \right)I_{solar}\left( \lambda\right)d\lambda\#\left( 4 \right) \end{aligned}$$

Here, $I_{solar}$ is local solar irradiance.

$P_{comb}\left( T_{s},T_{amb} \right)$is the input non-radiative power induced by the combined effects of heat convection and heat conduction, which can be expressed by:

$$\begin{aligned} P_{comb}\left( T_{s},T_{amb} \right)=h_{c}\left( T_{amb}-T_{s} \right)\#\left( 5 \right) \end{aligned}$$

Here, $h_{c}$ is the combined heat transfer coefficient due to heat convection and conduction of radiative cooler with local environment.

To obtain the radiative cooling power, we used the MATLAB to solve the equations. In our work, there is an overestimation of cooling power in experimental test with the measured cooling power of around 56.2 W/m^2^ and the simulated cooling power of around 42.9 W/m^2^. During our cooling power measurement, a polyimide film heater is attached to the bottom surface of aluminum plate, while the thermocouple is in between. Inevitably, there is an air gap between the aluminum plate and polyimide film heater due to the thermocouple wire, which could result in heating power loss due to the intrinsic thermal contact resistance. Moreover, the thermal conductivity of the thermal insulator (EPS) used in the experiment was ~0.03 W/m·K, leading to direct power loss from the heater to bottom interface. As a result, the abovementioned experimental errors could have resulted in the overestimation of cooling power.


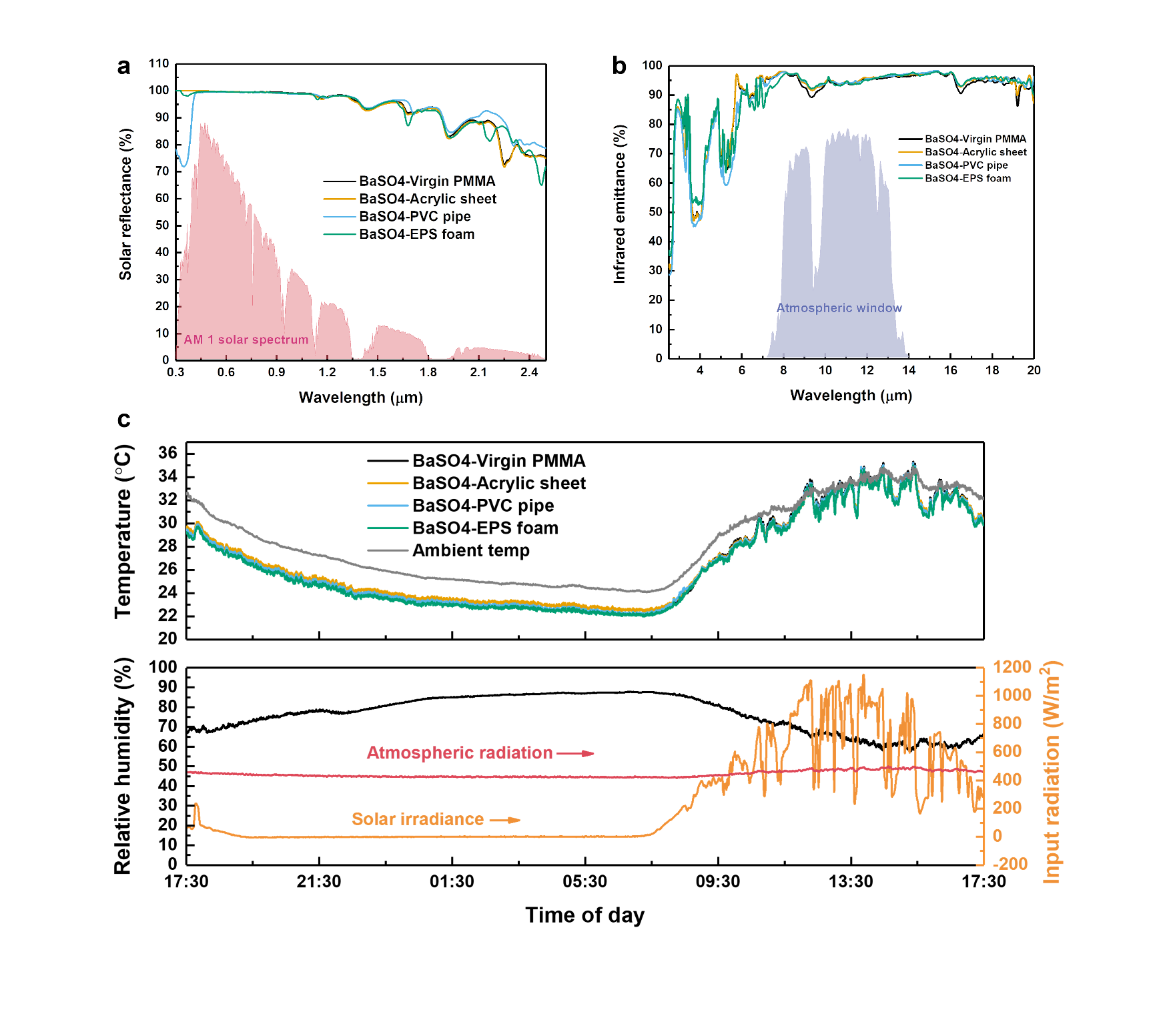


**Figure S2:** Optical and radiative cooling performance of the radiative cooling samples using sol-gel method. (a) Measured solar reflectance of the BaSO_4_ coating using the virgin PMMA, recycled acrylic sheet, (b) Measured infrared emittance of the BaSO_4_ coating using the virgin PMMA, recycled acrylic sheet, PVC pipe and EPS foam. (c) Measured surface temperature of related samples with local ambient air temperature, humidity, solar irradiance and atmospheric radiation (15-Jan-2022).


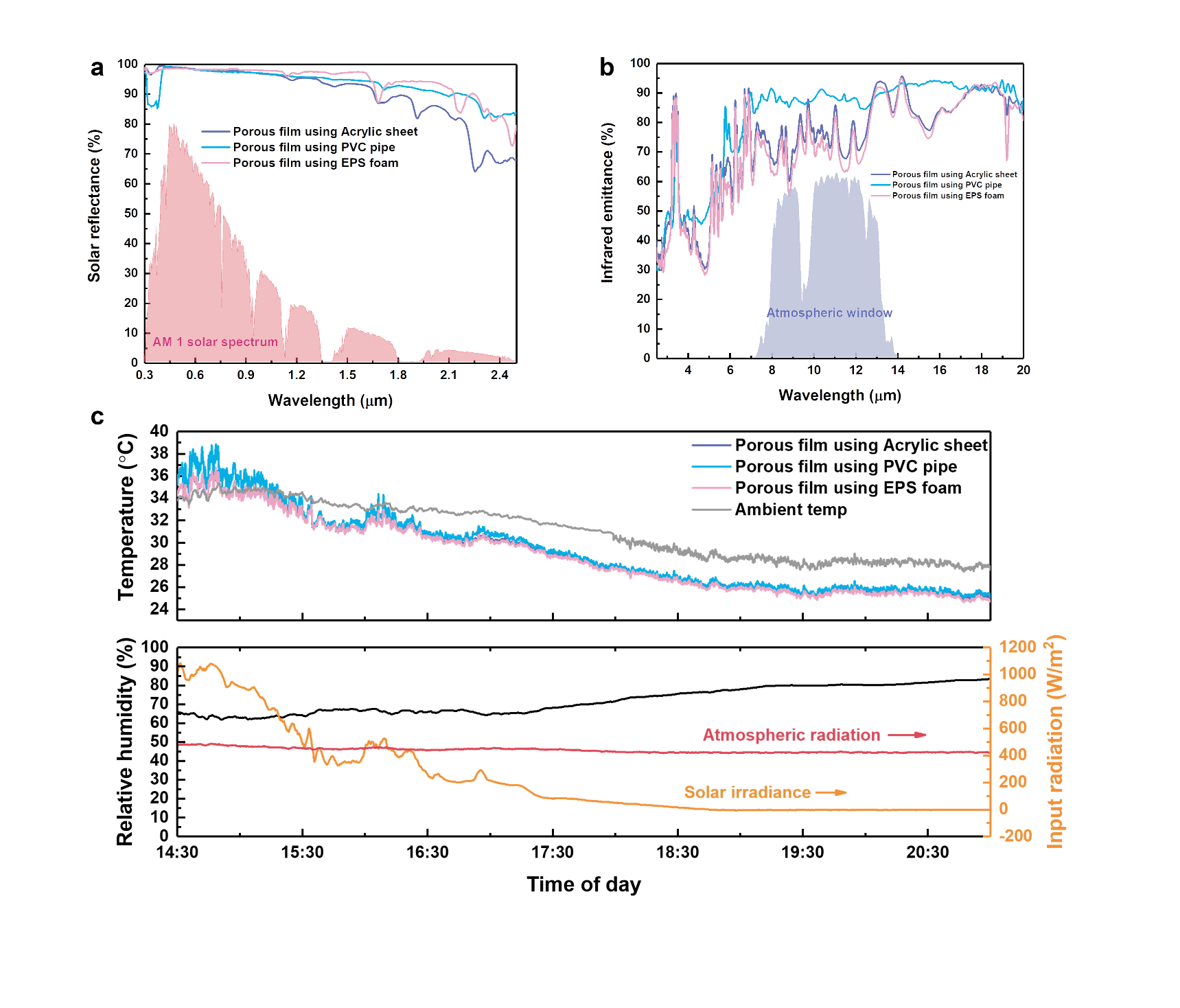


**Figure S3:** Optical and radiative cooling performance of the radiative cooling samples using phase inversion method. (a) Measured solar reflectance of the porous film using the recycled acrylic sheet, PVC pipe and EPS foam. (b) Measured infrared emittance of the porous film using the recycled acrylic sheet, PVC pipe and EPS foam. (c) Measured surface temperature of related samples with local ambient air temperature, humidity, solar irradiance and atmospheric radiation (27-Oct-2022).


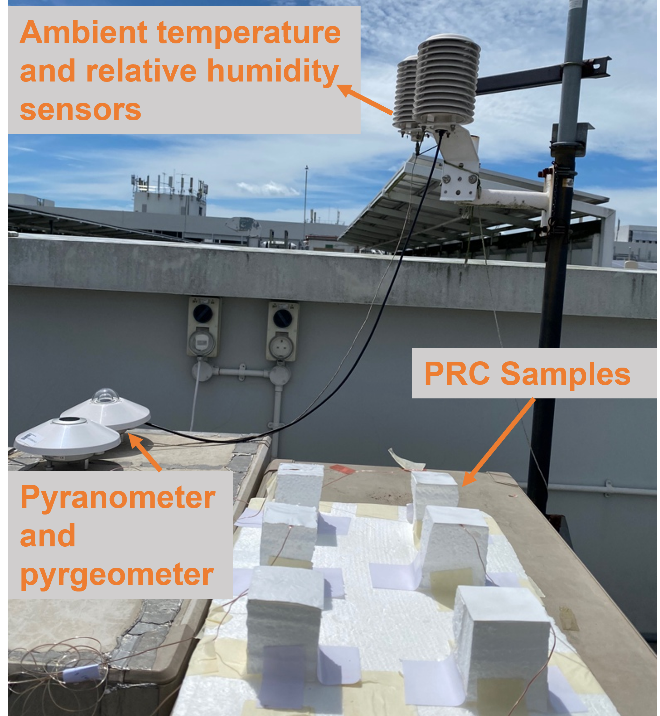


**Figure S4:** PRC samples and weather station for the cooling performance tests on the rooftop.

# References

[1] "MODTRAN, available from <http://modtran.spectral.com/modtran_home>." (accessed).

[2] C. G. Granqvist and A. Hjortsberg, "Radiative cooling to low temperatures: General considerations and application to selectively emitting SiO films," *J. Appl. Phys.,* vol. 52, no. 6, pp. 4205-4220, 1981.
